# Cyclin-dependent kinase-associated protein phosphatase is overexpressed in alcohol-related hepatocellular carcinoma and influences xenograft tumor growth

**DOI:** 10.3892/or.2012.2208

**Published:** 2012-12-24

**Authors:** WEY-RAN LIN, MING-WEI LAI, CHAU-TING YEH

**Affiliations:** 1 Department of Gastroenterology and Hepatology, Linkou Chang Gung Memorial Hospital, Taoyuan, Taiwan, R.O.C.; 2 Liver Research Center, Taipei Chang Gung Memorial Hospital, Taipei, Taiwan, R.O.C.; 3 Department of Pediatrics, Chang Gung Children's Hospital, Taoyuan, Taiwan, R.O.C.; 4 Chang Gung University College of Medicine, Taoyuan, Taiwan, R.O.C.

**Keywords:** hepatocellular carcinoma, cyclin-dependent kinase-associated protein phosphatase, xenograft tumor

## Abstract

The cyclin-dependent kinase (Cdk)-associated protein phosphatase (KAP) is a dual-specificity phosphatase that dephosphorylates Cdk2 and inhibits cell cycle progression. The overexpression of KAP has been found in breast, prostate and renal cell carcinomas. However, the role of KAP in hepatocellular carcinoma (HCC) remains unclear. Therefore, the aim of this study was to investigate the expression of KAP in HCC and elucidate its role in tumorigenesis. HCC tissues from 117 patients undergoing surgical resection were collected for western blot analysis and immunohistochemichal analysis to establish clinical correlation. The antisense-mediated inhibition of KAP expression was performed in Huh-7 cell lines for tumorigenicity and growth regulation experiments. Clinicopathological analysis indicated that KAP was overexpressed in HCC tissue from alcoholic patients (P<0.001). It was significantly overexpressed in patients with a tumor number of <3 (P=0.0271), suggesting the potential role of KAP in tumorigenesis during early-stage alcohol-related HCC. Additionally, the antisense-mediated inhibition of KAP in Huh-7 HCC cells interfered with cell cycle progression, decreased cell proliferation, reduced the colony-forming ability of the cells and increased apoptosis. Tumorigenicity experiments showed that the KAP knockdown in Huh-7 cells generated smaller tumors in nude mice compared with the mock controls (P=0.018). In the cells in which KAP had been knocked down, the physical interaction between KAP and Cdk2 significantly increased, despite the reduced expression levels of KAP. The phosphorylation of cell proliferation and apoptosis-associated proteins, including phosphatase and tensin homolog (PTEN), glycogen synthase kinase (GSK), p44/42 and Akt, was decreased. Therefore, it can be concluded that KAP is overexpressed in alcohol-related HCC. The antisense-mediated knockdown of KAP in Huh-7 cells decreased cell proliferation, reduced the colony-forming ability of the cells, interfered with cell cycle progression and suppressed xenograft tumor formation, partly through enhanced KAP and Cdk2 interaction.

## Introduction

Cyclin-dependent kinases (Cdks) and the cyclins are the major regulators of cell cycle progression. Their activities are controlled by a complex system directing stepwise phosphorylation and dephosphorylation events. For example, the activation of Cdk2 requires binding to cyclin A and the phosphorylation of Cdk2 at a conserved threonine, T160 (1,2). On the other hand, the phosphorylation of the other 2 conserved residues in the catalytic cleft (tyrosine 15 and threonine 14) inhibits the activity of activated Cdk2 (3,4). Such regulatory phosphorylation pathways are conserved among various species, from yeast to human (5). It has been shown that several kinases, such as Cdk7-cyclin H, a component of TFIIH in humans, and Cdk-activating kinase 1, a serine/threonine kinase in budding yeast, have the ability to phosphorylate T160 (6,7). On the other hand, the dephosphorylation of T160 can be achieved by various enzymes, such as protein phosphatase 2A and a dual-specific phosphatase, termed Cdk-associated protein phosphatase (KAP) (8,9). Since these enzymes can phosphorylate or dephosphorylate T160, they have been considered to play a role in regulating Cdk activity.

KAP, also known as cyclin-dependent kinase interactor 1 (Cdi1), is the product of the Cdk inhibitor 3 (*CDKN3*) gene (10–12). The expression of KAP increases at the G_1_-S phase transition. It forms stable complexes with Cdk2 and counteracts the stimulatory effect of Cdk-activating kinase on Cdk2 activity (9). It has been shown that the overexpression of KAP delays cell cycle progression in yeast and HeLa cells. On the other hand, the KAP-mediated dephosphorylation can be abolished by the binding of cyclin A to Cdk2, despite the fact that KAP can still bind to the cyclin A-Cdk2 complex. KAP can also bind to 2 other cell cycle regulators, Cdc2 and Cdk3. However, direct evidence is still lacking regarding the regulatory effect of KAP on these proteins (10,11).

The fact that KAP is one of the important regulators of cell cycle progression raises the possibility that it may participate in carcinogenesis. However, few reports have addressed the role of KAP in cancer and the results were somewhat contradictory. Our previous studies have demonstrated that various aberrant KAP mRNA transcripts, which encode truncated KAP mutants lacking phosphatase activity, can be found in hepatocellular carcinoma (HCC) and in cultured hepatoma cells (13). These truncated KAP mutants are capable of inhibiting protein interaction *in vitro* between wild-type KAP and Cdk2 (14). The aberrantly-spliced KAP transcripts have also been found in glioblastoma (15). In this type of cancer, aberrant splicing leads to the generation of a dominant-negative KAP variant that increases cell proliferation and tumor migration. By contrast, KAP has been reported to be overexpressed in breast and prostate cancers, suggesting a growth-promoting effect (16). Our recent study also demonstrated that the expression of KAP was associated with poorly differentiated human renal cell carcinoma and that the overexpression of KAP *in vitro* enhanced cell proliferation, resistance to apoptosis and xenograft tumor formation (17). However, these findings were difficult to explain, since they were in conflict with the established role of KAP in cell cycle inhibition.

HCC is the sixth most common cancer and the third most frequent cause of cancer-related mortality worldwide (18). More than 70% of HCCs develop within an established background of chronic liver disease. In eastern Asia, the dominant risk factor is chronic hepatitis B virus (HBV) infection, while in North America, Europe and Japan, hepatitis C virus (HCV) infection is the major risk factor, in conjunction with alcohol abuse (19). The mechanistic events in HCC carcinogenesis are complex and, to date, few molecular markers that correlate with the etiology and prognosis of cancer have been reported (20,21). Despite the aberrant KAP mRNA transcripts found in HCC, the role of KAP expression in HCC remains unclear. In this study, we investigated whether KAP expression correlates with clinicopathological factors in HCC, including etiology, pathological staging and clinical prognosis. Furthermore, the growth-regulatory effects of KAP in HCC were evaluated *in vitro* by antisense-mediated knockdown in Huh-7 cells. We aimed to elucidate the possible role of KAP in hepatocarcinogenesis.

## Materials and methods

### Patients and HCC tissues

Under the approval of the Institutional Review Board, Chang Gung Memorial Hospital, a total of 117 HCC patients undergoing surgical resection from January 1996 to January 2002 at Linkou Chang Gung Memorial Hospital, Taoyuan Hsien, Taiwan were included in this study. The samples were retrieved from the Chang Gung Tissue Bank. Clinicopathological information (including gender, age, etiology, pathological subtype, histological grading, biochemistries, tumor number and size) was retrospectively reviewed. All cancerous and non-cancerous liver samples derived from the periphery of the primary cancers were collected for analysis. Immunohistochemistry and western blot analysis were performed using mouse anti-KAP antibody (BD Biosciences, San Jose, CA, USA). Actin was detected using mouse anti-β-actin antibody (clone mAbcam 8226; Abcam Inc., Cambridge, MA, USA). The quantification of the tumor-to-non-tumor KAP expression ratio (T/N ratio) on the western blots was measured by ImageJ software (developed by NIH, USA).

### Plasmid construction, cell culture, transfection and establishment of stable transformants

To knock down KAP expression, a plasmid capable of producing an antisense transcript of KAP was constructed by inserting the *Eco*RI and *Bam*H1 (reverse) fragment of pB42AD-KAP (14) into the pcDNA 3.1/V5-His A (Clontech) *Bam*H1-*Eco*RI site. The construct, pcDNA-KAPr, was sequence-verified and transfected into human Huh-7 HCC cells. The cells were maintained in Dulbecco's modified Eagle's medium containing 10% fetal bovine serum (FBS) and stable clones were selected by neomycin (G-418, Geneticin; Invitrogen).

### Cell proliferation assay

Cell proliferation was assessed by the 3-(4,5-dimethylthiazol-2-yl)-2,5-diphenyltetrazolium bromide (MTT) assay. Cells were grown in a 96-well plate at an initial density of 5×10^3^ cells per well. On the day of the assay, cells were incubated at 37°C for 4 h in a culture medium containing 0.5 mg/ml MTT and then lysed by dimethyl sulfoxide (DMSO). The absorbance was measured by a spectrophotometer at 570 nm. Three independent experiments were performed for each measurement.

### FACS analysis of cell cycle

Cells were plated at a density of 2×10^5^ per 6-mm dish in complete medium for 24 h. 5-Bromo-2′-deoxy-uridine (BrdU) was diluted to a concentration of 1 mM with 1X Dulbecco's phosphate-buffered saline (DPBS) according to the BD Pharmingen BrdU Flow kit instruction manual. BrdU (10 μl/ml medium) was added to the plates (final concentration, 10 μM of BrdU) except for the unpulsed control plate. Cells were incubated at 37°C with 5% CO_2_ for 1 h. BrdU detection in the cells was performed with a fluorescein anti-BrdU antibody (BD Biosciences) according to the manufacturer's instructions.

### BrdU incorporation assay to determine DNA synthesis in the cell cycle

Cells were plated at a density of 1×10^4^ cells per well in a 96-well plate in serum-free DMEM for 48 h to synchronize the cells at the G_0_ phase. The medium was replaced with DMEM containing 10% FBS to initiate the cell cycle. Cells were then pulsed with 10 μM BrdU for 1 h at a 3-h interval from 0 to 24 h. The amount of incorporated BrdU at each time-point was measured by a chemiluminescence immunoassay, the BrdU Cell Proliferation ELISA (Roche Applied Science, Indianapolis, IN, USA) according to the manufacturer's instructions. All assays were carried out in triplicate.

### Terminal deoxynucleotidyl transferase dUTP nick end-labeling (TUNEL) assay for detection of apoptosis

The measurement of fragmented DNA from apoptotic cells by incorporating flourescein 12-dUTP at the 3′-OH DNA end, using the enzyme terminal deoxynucleotidyl transferase (TdT), was used to detect apoptosis. To perform this assay, cells in 5×10^4^ seeding density were plated on coverslips in a 12-well plate. Recombinant human tumor necrosis factor (TNF)-α (25 ng/ml; 2.8×10^7^ U/mg, R&D Systems, Minneapolis, MN, USA) and actinomycin-D (1 μg/ml, Boehringer Ingelheim, Mannheim, Germany) were then added to the growth medium. Cells were cultured for a further 8 h in a 37°C incubator and then fixed in 4% paraformaldehyde. The DeadEnd™ Fluorometric TUNEL System (Promega, Madison, WI, USA) was applied according to the manufacturer's instructions. The TUNEL-stained coverslips were mounted onto slides with Vectashield Mounting Medium with DAPI (Vector Laboratories, Inc., Burlingame, CA, USA) and immediately examined under a fluorescence microscope. The ratios of TUNEL-positive apoptotic cells to DAPI-positive cells in 10 random fields at ×200 magnification were calculated. The assay was carried out in triplicate.

### Colony-forming assay

Soft agar assay was performed in 6-well plates by growing 1×10^3^ cells per well in DMEM with 10% FBS and 0.35% low melting agarose on top of a 0.8% agarose base layer. The colonies were counted after staining with crystal violet 21 days following incubation.

### Tumorigenicity experiments in nude mice

Male athymic BALB/c nude mice were obtained from the National Animal Experimental Center (Taipei, Taiwan). The procedures for animal experiments were approved by our local (Linkou Chang Gung Memorial Hospital, Taoyuan, Taiwan) Animal Ethics Committee. The mice were maintained under specific pathogen-free conditions and used for the experiments when they reached 4 weeks of age. Cells were harvested by trypsinization and 1×10^6^ cells with >95% viability were injected subcutaneously into the right side of the backs of nude mice. Tumor formation was monitored daily until week 10. Tumor size was calculated as 1/2 × ab^2^, where a is the longest diameter and b is the shortest diameter of the tumor. Ten weeks later, the mice were sacrificed and the tumors were isolated for examination.

### Immunoprecipitation by anti-Cdk2

The Huh-7-pcDNA-KAPr and Huh-7-mock cells were lysed in 1 ml RIPA buffer [150 mM NaCl, 1.0% NP-40, 0.5% sodium deoxycholate, 0.1% sodium dodecyl sulfate, 50 mM Tris (pH 7.5), 1 mM phenylmethylsulfonyl fluoride (PMSF) and 10 g/ml leupeptin] for immunoprecipitation by anti-Cdk2 (Cdk2-Ab4, NeoMarkers, Fremont, CA, USA). The precipitate was then analyzed by polyacrylamide gel electrophoresis and subsequently electrotransferred onto a nitrocellulose membrane for western blot analysis. KAP was detected by mouse monoclonal anti-KAP antibody (BD Biosciences).

### Western blot analysis of proliferation-and apoptosis-related proteins

To investigate whether a growth regulatory signaling pathway was involved, western blot analysis was performed. The following antibodies were used: rabbit anti-phosphatase and tensin homolog (PTEN) antibody and anti-phospho-PTEN (Ser380) antibody (Cell Signaling Technology, Inc., Beverly, MA, USA); rabbit anti-Akt antibody (Abcam Inc.); rabbit anti-phospho-AKT (Ser473) antibody (Cell Signaling Technology, Inc.); rabbit anti-glycogen synthase kinase (GSK)3 antibody (Imgenex Corp., San Diego, CA); rabbit anti-phospho-GSK3 (Ser9) antibody (Cell Signaling Technology, Inc.); rabbit anti-p44/42 mitogen-activated protein kinase (MAPK) antibody (Cell Signaling Technology, Inc.); and rabbit anti-phospho-p44/42 MAPK (Thr202/Tyr204) antibody (Cell Signaling Technology, Inc.). Actin was detected using mouse anti-β-actin antibody (clone mAbcam 8226, Abcam Inc.).

## Results

### KAP expression in HCC

**Figure 1:**
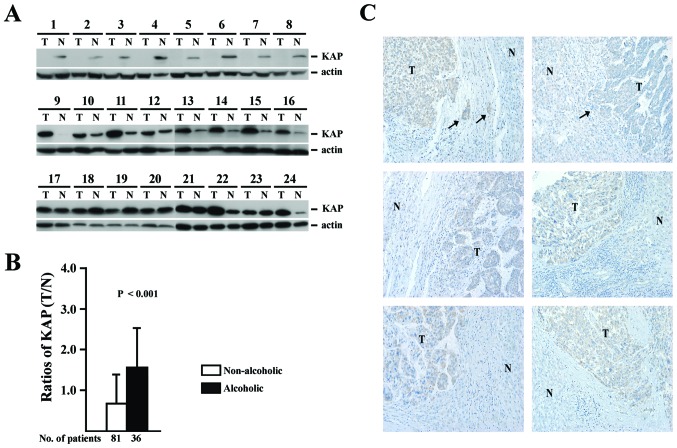
(A) Western blot analysis of KAP expression in HCC and non-HCC liver tissues from 24 patients. (B) T/N ratios of KAP expression. White bar shows that the mean ratio of 81 patients with non-alcoholic etiologies was <1. Black bar shows that the mean ratio of 36 patients with alcoholic etiology was >1 (P<0.001). (C) Immunohistochemical staining of alcohol-related HCC tissues. KAP was strongly expressed in the tumor compared to non-tumor sections. The arrows indicate the tumor cells surrounded by fibrotic bands.

To identify the expression pattern of KAP in HCC, 117 HCC tissue sampes obtained by surgical resection were subjected to western blot analysis for KAP expression. All patients had clinically localized disease and were treated with partial hepatectomy. Adjuvant chemotherapy and immunotherapy were not administered to the patients prior to surgery. The basic clinicopathological data are listed in Table I. Tissues obtained from tumor and non-tumor sections of the same patient were analyzed by western blot analysis, as shown in Fig. 1A. To investigate the association between KAP expression and clinicopathological parameters, the ratios of KAP expression in tumor and non-tumor sections (T/N ratios) were measured and correlated with clinicopathological parameters as well as histopathological features by univariate and multivariate statistical analysis, as shown in Table II. The histopathological features (including micro-and macrovascular invasion, Edmondson's histological grading, encapsulation, microsatellite lesions, tumor number and size) were not associated with the T/N ratios of KAP expression.

On the other hand, while most of the clinical parameters, including age, gender, cirrhosis, HBV surface antigen (HBsAg) serological positivity, anti-HCV serological positivity, ascites, α-fetoprotein (AFP), alanine aminotransferase (ALT) and Child-Pugh classification, were not associated with KAP expression, alcoholism and AST showed a positive correlation with increased KAP expression in the tumor sections (higher T/N ratios). However, since AST is usually higher in alcoholic patients, it was not associated with KAP expression following adjustment for the alcoholism factor (P<0.001). The T/N ratios of KAP between alcoholic and non-alcoholic patients are shown in Fig. 1B and KAP expression in the tumor sections was found to be significantly higher in HCC samples from alcoholic patients.

The association between KAP T/N ratios and tumor number in the 36 alcoholic patients is shown in Table III. An even higher expression of KAP was observed in the tumor sections from alcoholic HCC patients with <3 tumors. To confirm this finding, KAP immunohistochemistry staining was performed on HCC tissues from alcoholic HCC patients with <3 tumors, as shown in Fig. 1C. Again, a stronger staining for KAP was observed in the tumor sections compared to the adjacent non-tumor sections. The finding that KAP expression was significantly increased in the tumor sections of alcoholic HCC patients with a smaller number of tumors (<3) suggested that KAP may play a role in alcohol-related carcinogenesis.

### Antisense-mediated suppression of KAP in Huh-7 cells results in decreased proliferation and increases apoptosis

**Figure 2:**
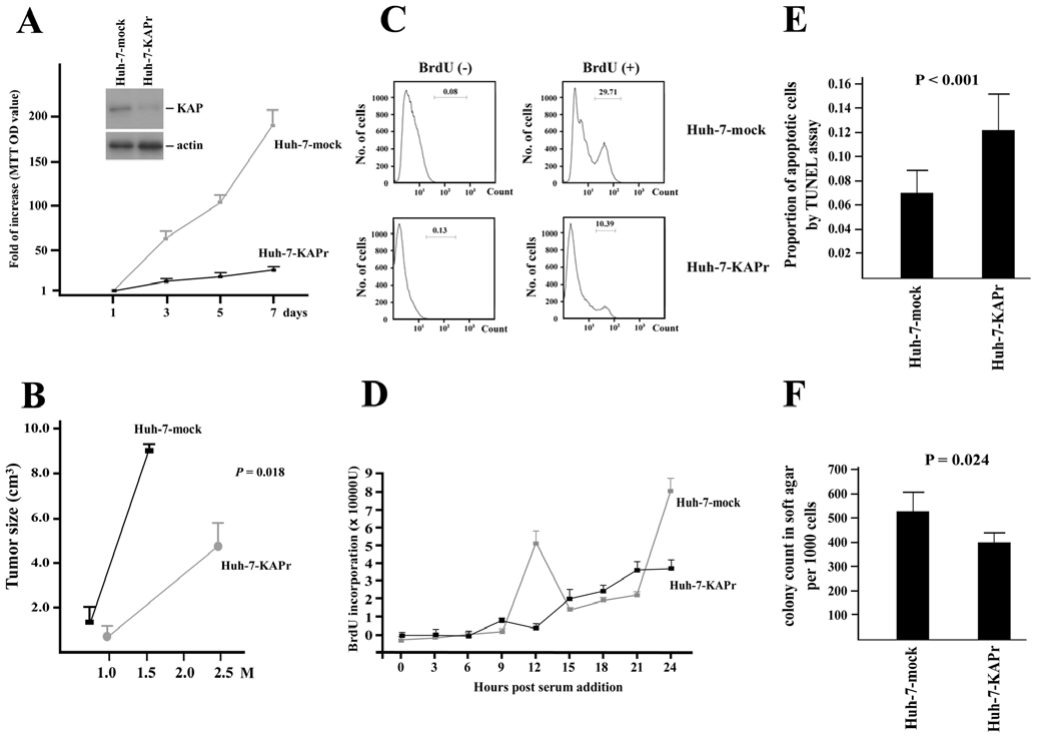
(A) Cell proliferation was assessed by MTT assays for mock-transfected (gray square) and KAPr transfected (black square) cells. (B) Tumorigenicity assay in nude mice. Mock-transfected (black square) and KAPr-transfected (gray circle) cells were injected subcutaneously into the backs of nude mice and tumor size was assessed at 6 and 10 weeks, respectively. (C) Cell cycle analysis by FACS. The number of BrdU(+) cells was higher in the Huh-7-mock than Huh-7KAPr cells. (D) Cell cycle time assessment of Huh-7-mock (gray square) and Huh-7-KAPr (black square) cells by BrdU incorporation. The peaks of BrdU in Huh-7-mock cells were shown at 12 and 24 h, while these peaks were not observed in Huh-7-KAPr cells. (E) Cell apoptosis was assessed by TUNEL assay. There was a higher number of apoptotic cells among the Huh-7-KAPr cells than among the Huh-7-mock cells (P<0.001). (F) The colony-forming ability of the Huh-7-mock and Huh-7-KAPr cells was assessed by soft agar colony formation assay. The Huh-7-mock cells had a higher colony count compared to the Huh-7-KAPr cells (P=0.024).

To evaluate the role of KAP expression in HCC cells, KAP knockdown was achieved by the transfection of pDR2-KAPr, which expressed an antisense KAP RNA fragment. Western blot analysis of KAP expression showed a significant decrease in KAP expression in the Huh-7-KAPr cells compared to the Huh-7-mock cells, as shown in Fig. 2A. MTT cell proliferation assays showed a decreased cell growth rate in the Huh-7-KAPr cells (Fig. 2A). To confirm the association between KAP and the cell proliferation of Huh-7 cells, a FACS analysis of BrdU expression in the Huh-7-KAPr and Huh-7-mock cells was performed and the results are shown in Fig. 2C. The number of BrdU-positive cells was significantly decreased among the Huh-7-KAPr cells compared to the Huh-7-mock cells, suggesting that the inhibition of KAP expression resulted in decreased cell proliferation.

In order to determine whether KAP affected cell apoptosis, TUNEL assay was performed on the Huh-7-KAPr and Huh-7-mock cells. The proportion of apoptotic cells was significantly higher among the Huh-7-KAPr cells, suggesting that the inhibition of KAP enhanced Huh-7 cell apoptosis (Fig. 2E).

### Inhibition of KAP in Huh-7 cells affects normal cell cycle progression

The Huh-7-KAPr and Huh-7-mock cells were synchronized by serum starvation for 48 h. The amount of BrdU incorporation was assessed every 3 h after the addition of FBS to the culture medium. The first peak of BrdU incorporation was detected 12 h after cell cycle initiation in the Huh-7-mock cells, while no obvious peak was observed in the Huh-7-KAPr cells within 24 h, suggesting that the KAP knockdown affected the normal cell cycle progression in the Huh-7 cells (Fig. 2D).

### Soft agar colony formation assay and tumorigenicity experiments

To investigate whether the anti-proliferation, apoptosis enhancement and cell cycle disturbance caused by KAP knockdown affected the colony-forming ability of the cells, the soft agar colony formation assay was performed. As shown in Fig. 2F, the Huh-7-KAPr cells exhibited decreased colony-forming ability compared to the Huh-7-mock cells. To further demonstrate the tumorigenic ability *in vivo*, the mock-transfected cells and the Huh-7-KAPr cells were injected subcutaneously into nude mice. The xenograft tumors grew quickly in the 6 weeks following the injection of Huh-7-mock cells, while the tumor size was significantly smaller even at 10 weeks following the injection of Huh-7-KAPr cells (8.9±0.2 cm^3^ vs. 4.3±1.3 cm^3^; P=0.018) (Fig. 2B).

### Increased Cdk2-KAP binding ability in Huh-7-KAPr cells

**Figure 3:**
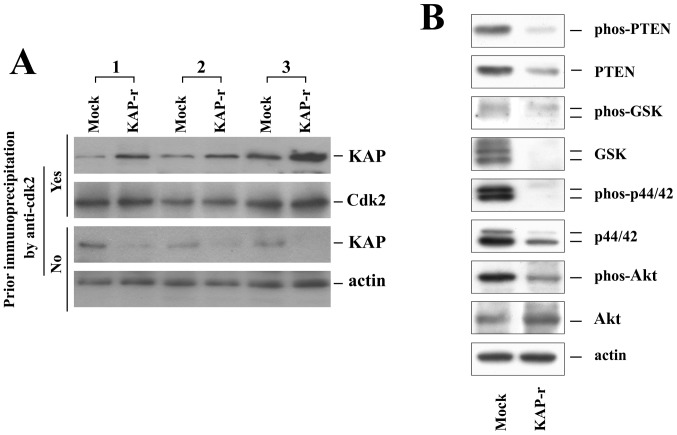
(A) KAP expression following immunoprecipitation with anti-Cdk2 antibody. The 3 independent experiments showed that KAP expression was higher in the Huh-7-KAPr cells than in the Huh-7-mock cells following immunoprecipitation with anti-Cdk2 antibody. (B) Expression of PTEN, GSK, p44/42, Akt and their phosphorylated forms in the Huh-7-mock and Huh-7-KAPr cells.

Cdk2 is the essential protein for the cell cycle G_1_/S phase transition. It is not only regulated by the regulatory cyclin subunits but also by phosphorylation. Binding to KAP can inactivate Cdk2 and disturb the cell cycle. Since cell proliferation was decreased in Huh-7-KAPr cells, we examined the Cdk2-KAP binding ability of the Huh-7-mock and Huh-7-KAPr cells by immunoprecipitation (Fig. 3A). Without prior immunoprecipitation by an anti-Cdk2 antibody, the KAP protein was expressed in smaller amounts in the Huh-7-KAPr cells than in the Huh-7-mock cells, as shown by the western blot analysis results. With prior co-immunoprecipitation by the anti-Cdk2 antibody, the amount of KAP protein was significantly higher in the Huh-7-KAPr than in Huh-7-mock cells in all 3 independent experiments. These results suggested that, despite the smaller amount of KAP protein produced by Huh-7-KAPr cells, the Huh-7-KAPr cells exhibited a stronger Cdk2-KAP binding ability.

### KAP affects PTEN, GSK, p44/42 and Akt activities

To determine whether KAP affects proteins associated with cell proliferation and apoptosis, PTEN, GSK, p44/42, Akt and their phosphorylated forms were analyzed in the Huh-7-mock and Huh-7-KAPr cells (Fig. 3B). While the expression of Akt was increased, the expression of PTEN, GSK and p44/42 was decreased in the Huh-7-KAPr cells. All the active phosphorylated forms of PTEN, GSK, p44/42 and Akt were decreased in the Huh-7-KAPr cells, suggesting that the activity of these proteins was suppressed in the Huh-7-KAPr cells.

## Discussion

In this study, KAP was overexpressed mainly during the early stages of alcohol-related HCC. The major risk factors of HCC include chronic HBV and HCV infection, exposure to aflatoxins and alcohol abuse. It is generally believed that HCC develops in the cirrhotic liver resulting from chronic inflammation and fibrosis caused by the above-mentioned factors (22). However, some unique mechanisms have been demonstrated which contribute to the development of HCC, specifically in patients with alcoholic liver disease (23,24). Acetaldehyde, the oxidized form of alcohol, has been identified as a toxic compound with mutagenic properties (25). It can form DNA adducts, such as N^2^-ethyl-2′deoxyguanosine (N^2^-ethyl-dG) and 1,N^2^-propano-2′-deoxyguanosine (1,N^2^-PdG), resulting in alterations in DNA integrity. Furthermore, chronic alcohol consumption causes the induction of cytochrome P450 2E1 (CYP2E1), which leads to the increased generation of acetaldehyde and reactive oxygen species (ROS) (26,27). Furthermore, CYP2E1 also metabolizes several toxic substrates, including pro-carcinogenic compounds in alcoholic beverages. The induction of CYP2E1 promotes carcinogenesis not only through its own CYP2E1-dependent metabolism, but also through affecting the rate of metabolism of other substrates (28–30).

Long-term alcohol consumption has been demonstrated to decrease hepatocyte growth *in vitro*(31,32) and hepatic regeneration following surgical ablation *in vivo*(33,34). It has also been demonstrated that alcohol consumption upregulates the expression and activity of guanine nucleotide regulatory proteins (Gi-proteins) (35). The Gi-protein activation further increases MAPK activity and cellular mitogenesis in HCC. Despite these findings, the actual role of alcohol in HCC carcinogenesis is not yet completely understood. In this study, we demonstrate that KAP is overexpressed in alcohol-related HCC, particularly in patients with <3 tumors, suggesting a potential role of KAP in early alcohol-related carcinogenesis.

The studies on the role of KAP in carcinogenesis have been somewhat contradictory. Initially, it was suggested that KAP is an inhibitor of cell cycle progression and thus inhibits cell growth (9). However, ensuing studies demonstrated that KAP was overexpressed in breast, prostate and renal cell cancers, suggesting a growth-promoting role (16,17). Despite the fact that dominant-negative mutant transcripts of KAP have been identified in HCC and glioblastoma, these findings are not sufficient to explain why KAP overexpression promotes tumor cell growth. In this study, we found that the suppression of KAP in HCC *in vitro* resulted in decreased proliferation and reduced colony-forming ability of the cells, changes in the cell cycle, increased apoptosis and decreased tumorigenicity. These findings support those from previous studies on breast, prostate and renal cell carcinoma, which suggested that KAP plays a growth-promoting role in cancer. Our results also demonstrated that the phosphorylation of proliferation- and apoptosis-related proteins, including PTEN, GSK, p44/42 and Akt, was decreased in the Huh-7-KAPr cells, suggesting that the decrease in KAP expression suppressed the activity of these proteins and affected cell proliferation and apoptosis. Furthermore, our data showed that the Cdk2-KAP binding ability was increased in the KAP-suppressed HCC cells. The increased Cdk2-KAP binding ability may theoretically result in the decrease in the levels of phosphorylated Cdk2, leading to the inhibition of cell proliferation. However, the reason why the binding ability was increased remains unknown and further studies are warranted to resolve this issue.

In conclusion, our study demonstrates that KAP is overexpressed mainly during the early stages of alcohol-related HCCs. The suppression of KAP expression resulted in decreased cell proliferation, changes in the cell cycle, reduced colony-forming ability of the cells and the suppression of tumorigenicity, at least partly through the increased physical interaction with Cdk2 and thus the reduction of the active forms of growth-related proteins.

## Figures and Tables

**Table I:** Basic clinicopathological characteristics of the 117 HCC patients.

Parameter	Value
Age (years)	54.2±15.0^a^
Gender (male/female)	90/27
Cirrhosis	48
HBsAg-positive	80
Anti-HCV-positive	31
Tumor number	
1	76
2	15
3	20
4	6
Size (diameter, cm)	7.0±4.7^a^
Microvascular invasion	39
Edmondson's grading	
1–2	29
3	70
4	18
Encapsulation	86
Macrovascular invasion	0
Ascites	9
α-fetoprotein (ng/ml)	14 (3–327500)^b^
Albumin (g/dl)	3.8±0.7^a^
Bilirubin (mg/dl)	1.9±1.9^a^
Prothrombin time (sec)	12.3±1.4^a^
Creatinine (mg/dl)	1.2±1.5^a^
AST (U/l)	100.8±128.3^a^
ALT (U/l)	82.3±104.6^a^
Alcoholism	36
Time to last follow-up or death (months)	25 (2–127)^b^

a Mean ± SD;

b Median (range).

HBsAg, hepatitis B surface antigen; anti-HCV, antibody against hepatitis C virus; AST, aspartate aminotransferase; ALT, alanine aminotransferase

**Table II:** Univariate and multivariate analysis of clinicopathological parameters associated with T/N ratio of KAP in HCC.

Factors	Groups	Pt. no. (Mean ± SD)	KAP T/N ratio (95% CI)	Unadjusted β (95% CI)	Adjusted β
Gender	Male	90	1.05±0.89	0.28 (−0.09, 0.65)	
	Female	27	0.77±0.71		
Age (years)	>54	62	0.90±0.81	−0.19 (−0.50, 0.12)	
	≤54	55	1.09±0.91		
Cirrhosis	Yes	48	1.03±0.99	0.74 (−0.25, 0.39)	
	No	69	0.96±0.76		
HBsAg	Positive	80	1.02±0.86	0.11 (−0.22, 0.45)	
	Negative	37	0.91±0.85		
Anti-HCV	Positive	31	1.10±1.05	0.15 (−0.21, 0.50)	
	Negative	86	0.95±0.78		
Microvascular invasion	Yes	39	1.12±1.00	0.21 (−0.13, 0.54)	
	No	78	0.92±0.77		
Macrovascular invasion	Yes	9	1.26±0.75	0.30 (−0.29, 0.89)	
	No	108	0.97±0.86		
Edmondson's histological grading	>II	87	0.94±0.83	−0.19 (−0.55, 0.17)	
	≤II	30	1.13±0.93		
Capsule	Yes	86	0.95±0.75	−0.16 (−0.52, 0.19)	
	No	31	1.11±1.10		
Microsatellite lesions	Yes	20	1.25±0.88	0.32 (−0.10, 0.73)	
	No	97	0.93±0.85		
Tumor number	>1	41	0.95±0.88	−0.95 (−0.43, 0.24)	
	1	74	1.04±0.85		
Size (cm)	>7.0	37	1.02±0.67	0.01 (−0.33, 0.36)	
	≤7.0	77	1.00±0.94		
Ascites	Yes	9	1.13±0.76	0.16 (−0.43, 0.75)	
	No	108	0.98±0.87		
AFP (ng/ml)	>400	31	0.79±0.75	−0.27 (−0.62, 0.09)	
	≤400	86	1.06±0.89		
AST (U/l)	>100	32	1.30±1.00	0.43 (0.08, 0.77)	0.00 (−0.00, 0.00)^a^
	≤100	85	0.87±0.77		
ALT (U/l)	>80	31	1.18±0.77	0.19 (−0.17, 0.55)	
	≤80	86	0.94±0.88		
Alcoholism	Yes	36	1.58±0.93	0.86 (0.56, 1.16)	0.87 (057, 1.18)^b^
	No	81	0.72±0.68		
Child-Pugh classification	A	100	0.96±0.82	−0.20 (−0.64, 0.25)	
	B	17	1.16±1.06		

Pt. no., patient number; HBsAg, hepatitis B surface antigen; anti-HCV, antibody against hepatitis C virus; AFP, α-fetoprotein; AST, aspartate aminotransferase; ALT, alanine aminotransferase; CI, confidence interval.

a 0.001 (−0.0003, 0.002); P=0.187;

b P<0.001.

**Table III:** Association betwen KAP T/N ratios and tumor number in the 36 alcoholic HCC patients.

Tumor number	No. of patients	KAP T/N ratios
<3	26	1.21±0.38^a^
≥3	10	0.90±0.29^a^

a P=0.0271.
